# Identification of Coffee Varieties Using Laser-Induced Breakdown Spectroscopy and Chemometrics

**DOI:** 10.3390/s18010095

**Published:** 2017-12-31

**Authors:** Chu Zhang, Tingting Shen, Fei Liu, Yong He

**Affiliations:** 1 College of Biosystems Engineering and Food Science, Zhejiang University, 866 Yuhangtang Road, Hangzhou 310058, China; chuzh@zju.edu.cn (C.Z.); ttingshen@zju.edu.cn (T.S.); yhe@zju.edu.cn (Y.H.); 2 Key Laboratory of Spectroscopy Sensing, Ministry of Agriculture, Hangzhou 310058, China

**Keywords:** coffee varieties, laser-induced breakdown spectroscopy, discrimination, chemometrics, wavelength selection

## Abstract

We linked coffee quality to its different varieties. This is of interest because the identification of coffee varieties should help coffee trading and consumption. Laser-induced breakdown spectroscopy (LIBS) combined with chemometric methods was used to identify coffee varieties. Wavelet transform (WT) was used to reduce LIBS spectra noise. Partial least squares-discriminant analysis (PLS-DA), radial basis function neural network (RBFNN), and support vector machine (SVM) were used to build classification models. Loadings of principal component analysis (PCA) were used to select the spectral variables contributing most to the identification of coffee varieties. Twenty wavelength variables corresponding to C I, Mg I, Mg II, Al II, CN, H, Ca II, Fe I, K I, Na I, N I, and O I were selected. PLS-DA, RBFNN, and SVM models on selected wavelength variables showed acceptable results. SVM and RBFNN models performed better with a classification accuracy of over 80% in the prediction set, for both full spectra and the selected variables. The overall results indicated that it was feasible to use LIBS and chemometric methods to identify coffee varieties. For further studies, more samples are needed to produce robust classification models, research should be conducted on which methods to use to select spectral peaks that correspond to the elements contributing most to identification, and the methods for acquiring stable spectra should also be studied.

## 1. Introduction

Coffee is one of the most popular beverages in the world. According to the International Coffee Organization (ICO), the estimated global coffee consumption in 2014 was 149.2 million bags (at 60 kg per bag) [1]. Coffee variety is one of the key factors that influences coffee quality. The identification of coffee beans has been studied with traditional laboratory-based chemical methods [2,3,4] and spectroscopic techniques [5,6,7,8,9,10]. Spectroscopic techniques have been widely studied due to their advantages of speed and few, or no sample preparation, and being both easy to operate and contactless. Near-infrared (NIR) spectroscopy, mid-infrared (MIR) spectroscopy, nuclear magnetic resonance (NMR) spectroscopy, and Raman spectroscopy have been used in the coffee industry to study coffee authentication, variety, producing area, and quality determination. These methods focus on the samples’ chemical group and showed satisfactory results in the coffee industry [5,6,7,8,9,10].

Different samples have different kinds of elements with different contents. In many cases, element detection, such as element identification, element-content determination, and other related fields, is necessary. Laser-induced breakdown spectroscopy (LIBS) is a spectroscopic technique that identifies the elemental composition of samples [11]. LIBS analyzes the spectral emission from laser-induced plasmas by focusing a pulse laser beam on the sample’s surface. LIBS then identifies the spectral peaks and the spectral intensity corresponding to the element in the sample. LIBS can detect almost all elements, while simultaneously analyzing all those samples’ elements that are above the detection limit. When compared with traditional element measurement methods such as atomic absorption spectrometry (AAS), inductively coupled plasma mass spectrometry (ICP-MS), and inductively coupled plasma optical emission spectrometry (ICP-OES), LIBS offers the advantages of little or no sample preparation, along with a fast, simple, and relatively low-cost analysis.

LIBS is very popular in element detection because of its ability to detect almost all of them. LIBS has been used for qualitative and quantitative element detection for soil [12,13], plants [14,15], food [16,17], minerals [18,19], and planets like Mars [20,21,22]. In recent years, studies have focused on the establishment of LIBS as an effective detecting technique within the coffee industry. Gondal et al. analyzed the LIBS spectrum of a coffee sample, revealing the presence of Mg, Ca, Al, Cu, Na, Ba, Br, Co, Cr, Ce, Mn, and Mo [23]; Nufiqurakhmah et al. used the LIBS technique to identify the elemental contents of coffee beans and selected Ca, W, Sr, Mg, and H to differentiate regular and luwak beans from the Arabica variant [24]; Ferreira et al. attempted to classify roasted and ground coffee with low-cost LIBS devices and obtained results that showed accuracies above 84% for ensemble methods in some spectral ranges [25]; Anggraeni et al. used the LIBS technique to differentiate green coffee beans from Arabica and Robusta variants, and they confirmed that Ca, W, Mg, Be, Na, and Sr were element identifiers of green coffee beans [26]. The above articles show that LIBS is a promising technique for detecting coffee products. However, their qualitative analysis methods lack optimization and the accuracy rates have yet to be improved.

The LIBS technique generates a large amount of data, including background and spectral peaks that are element-related. The simplest use of LIBS serves to identify elements by observing spectral peaks. Dealing with LIBS spectra by extracting useful information for further qualitative and quantitative analysis is far more complex [27,28,29]. Chemometric methods are widely used to handle spectral data in different spectroscopy techniques, and researchers are now trying to improve the accuracy of qualitative and quantitative analysis using LIBS spectra. The qualitative analysis of LIBS spectra focuses on identification and classification, and the quantitative analysis focuses on element-concentration determination [27,28,29]. By extracting useful information and building qualitative and quantitative models, chemometric methods can be used to reduce the errors generated by random factors and to reveal sample differences caused by different factors. Preprocessing LIBS spectra, which includes baseline correction, noise reduction, normalization, outlier removal, and overlapping-peaks identification, is important for the reduction of errors caused by random factors such as instruments, the environment, and the operation [27,28,29,30,31,32,33,34]. A series of supervised (partial least squares-discriminant analysis (PLS-DA), artificial neural network (ANN), support vector machine (SVM), etc.) and unsupervised (principal component analysis (PCA), hierarchical cluster analysis (HCA), etc.) chemometric methods have been widely introduced as ways of handling LIBS data for qualitative analysis [27,28,29]. To quantitatively determine element concentrations, univariate and multivariate methods are being introduced in order to build regression models [27,28,29]. Although chemometric methods have proven to be effective, their adoption in LIBS spectra analysis still poses a great challenge.

The objective of this study was to identify coffee varieties using LIBS with chemometric methods. Our specific objectives were: (1) to identify the main elements in coffee; (2) to reduce noise by wavelet transform; (3) to select those elements that contribute most to coffee-variety identification; and (4) to build calibration models for coffee-variety identification using chemometric methods.

## 2. Materials and Methods

### 2.1. Sample Preparation

We collected four coffee-bean cultivars from China, including Typica Arabica coffee from Yunnan Province, Catimor Arabica coffee from Yunnan Province, Fushan Robusta coffee from Hainan Province, and Xinglong Robusta coffee from Hainan Province. For each variety, we ground twenty coffee beans, which we then mixed thoroughly as a sample and stored in a plastic bag. The coffee powders were pressed into a tablet by a tablet-press machine (FY-24, SCJS, Tianjin, China). In total, 24 samples from each variety were prepared. The Typic Arabica coffee from Yunnan Province, Catimor Arabica coffee from Yunnan Province, Fushan Robusta coffee from Hainan Province, and Xinglong Robusta coffee from Hainan Province were assigned, respectively, the category values 1, 2, 3, and 4.

### 2.2. LIBS System

The experimental system used in this study consists of a Q-switch Nd:YAG nanosecond pulsed laser (Vlite-200, Beamtech, Beijing, China). A second harmonic laser (532 nm, pulse duration of 8 ns, beam diameter of 7 mm) was used to ignite the sample with the help of a plano-convex lens (f = 50 mm). A detection system, consisting of an Echelle spectrograph (ME5000, Andor, Belfast, UK) and an intensified charge-coupled device (ICCD) camera (DH334, Andor), was used to collect plasma emission spectra in the range of 230 to 880 nm. The samples were placed on a X-Y-Z translation stage.

### 2.3. LIBS Data Acquisition

Before measurement, 0.5 g of coffee powder from each sample was pressed into a 15-mm diameter pellet for 60 s at a pressure of 12 tons. In order to improve the signal-to-noise rate, the delay and integration times were optimized to 1.8 μs and 5 μs, respectively. The laser was fired with a pulse energy of 60 mJ at 1 Hz. The spot size of the focused laser beam was 600 μm. For each sample, ten successive spectra were accumulated at each location, and 16 different locations were measured in ambient air with the help of the translation stage.

### 2.4. Chemometric Methods

We frequently used wavelet transform (WT) as a spectral-smoothing method. WT decomposes the original spectral data into its high-frequency and low-frequency parts. The noise was generally presented as the high-frequency part; we used WT for spectral-noise reduction by dealing with this high-frequency part via a threshold method. The preprocessed high-frequency and low-frequency parts were then reconstructed as the preprocessed spectra [35,36,37].

Principal component analysis is a widely used qualitative method in spectral data analysis. PCA transforms the original variables into new variables, which are linear combinations of the original variables. These new variables (called principal components) are orthogonal and ranked according to the explained variances. The first principal component (PC) explained most of the variances, and was followed by the second PC, the third PC, and so on. Generally, the first few PCs explained most of the variances. The score biplots, based on two different PCs, are commonly used to present the sample distributions in a score space [38].

Partial least squares-discriminant analysis is a supervised pattern recognition method based on partial least squares regression (PLSR). The PLSR explores the linear relationship between the spectral data and the corresponding features (physical, chemical, and category). PLSR transforms the original data into the new orthogonal variables (called latent variables (LVs)). The first few LVs that carried the most information were used for calibration. The PLS-DA uses the integer that represents the category instead of the chemical or physical features. The PLS-DA conducts the regression procedure; the prediction value of PLS-DA was a real number with decimals. A threshold value should be set to determine the category which the sample belongs to. In this study, the threshold value was set to 0.5 [39], and leave-one-out cross validation was conducted in order to obtain an optimal number of LVs.

Radial basis function neural network (RBFNN) is a widely used 3-layer feed-forward neural network. It can approximate any nonlinear function. RBFNN has a good generalization ability and a fast convergence speed. RBFNN creates a direct connection between the input layer and the hidden layer while connecting the hidden layer and the output layer via output weights [40]. The optimal spread value should be determined for RBFNN. In this study, we built RBFNN models using spread values from 0.1 to 1 with a step of 0.1, and from 1 to 100 with a step of 1. The optimal spread value was determined by the RBFNN model with the highest classification accuracy.

A support vector machine is a supervised discriminant method. It maps original data into a higher dimension space, and constructs hyperplanes that have a maximum distance to the nearest sample of a given category. The samples were then classified via the hyperplanes. Kernel functions are important in establishing SVM models, and radial basis function (RBF) is a widely used kernel function. The penalty coefficient (C) and kernel function parameter (γ) should be determined [41]. In this study, a grid-search procedure was used to determine the optimal combination of C and γ. The search range for C and γ ranged from 2⁻^8^ to 2^8^. The optimal combination of C and γ was determined by the SVM model with the highest classification accuracy.

### 2.5. Model Evaluation and Software

The performances of the classification models were evaluated via the classification accuracy of the calibration and prediction sets, defined as the percentage of correctly classified samples taken from all the samples. The PLS-DA, RBFNN, and SVM models and the PCA analysis were conducted on the Matlab R2010b (The Math Works, Natick, MA, USA).

## 3. Results

### 3.1. LIBS Spectra Preprocessing

Once the LIBS spectra were acquired and ready to be analyzed, the spectral data needed to be preprocessed to minimize both the influence of noise and the variations caused by the matrix effects, the experimental conditions, the sample status, and the LIBS system.

Given that the spectral intensity of some wavelengths had negative values due to spectral noise, a simple baseline correction was applied to the spectral data in order to eliminate the negative values. After this baseline correction, the LIBS spectra were preprocessed by WT to reduce the noise. The LIBS spectra were generally disturbed by the noise caused by the environment, the sample, and the instruments. The reduction of spectra noise was meant to enhance the signal-to-noise ratio, which is important in spectral data analysis. WT was an efficient method for reducing noise and keeping the spectral features. Its use consisted in the decomposition of the original data and the reconstruction of the preprocessed data. In this study, we used WT with a wavelet basis function Daubechies 5 (db5) and a decomposition level 10, after trials. Given that the spectra in the range of 229.98–246.51 nm had no obvious spectral peaks, we only analyzed the 246.53–880.26 nm spectra (20,937 variables).

**Figure 1:**
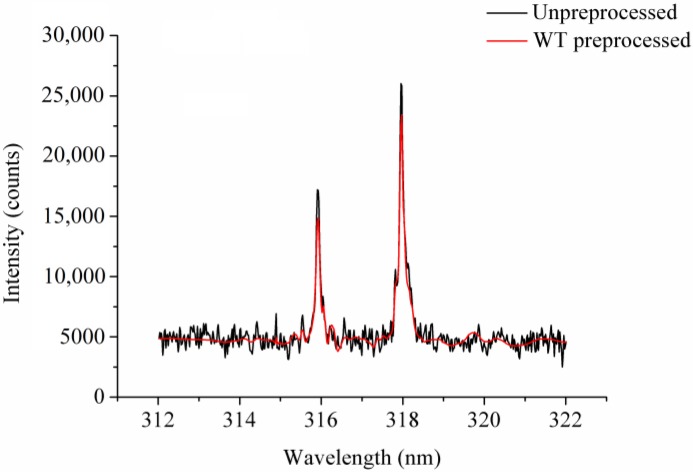
The un-preprocessed spectrum and the spectrum preprocessed by wavelet transform (WT).

Figure 1 shows the raw and the preprocessed 312.02–322.02 nm spectra of a randomly selected spectrum, where a small peak occurred. We observed that the WT-preprocessed spectrum was much smoother than the raw spectrum. However, the preprocessed spectrum’s peak intensities were slightly lower than the raw spectrum’s. The results show that WT reduced the noise efficiently, as the peaks were kept.

An efficient method for us to deal with the variations caused by matrix effects and experimental conditions was to use normalization [22]. In this study, the LIBS spectra were normalized [22] after being preprocessed by WT. The total areas under the 246.53–880.26 nm spectrum were calculated, and each spectrum was normalized to the total area.

Each sample’s LIBS spectra were collected from 16 of the sample’s sites. Due to the site-to-site variations, each site’s spectrum showed differences, and some of the spectra with larger variations were treated as outliers [22]. To remove the spectra with larger variations, the relative standard deviation (RSD) of the C I 247.86 nm intensity was calculated for each sample. In our study, the rule was to keep 75% of the original spectra of each variety after removing outliers [22]. To identify outliers, 75% of the original spectra were randomly selected to compute the RSD, a procedure that was repeated 100,000 times. The spectra with minimum RSDs were kept for further analysis, and the remaining 25% were identified as outliers. Thus, a total of 1152 spectra (288 from each cultivar) were selected for further analysis after outlier removal.

### 3.2. Features of LIBS Spectra

**Figure 2:**
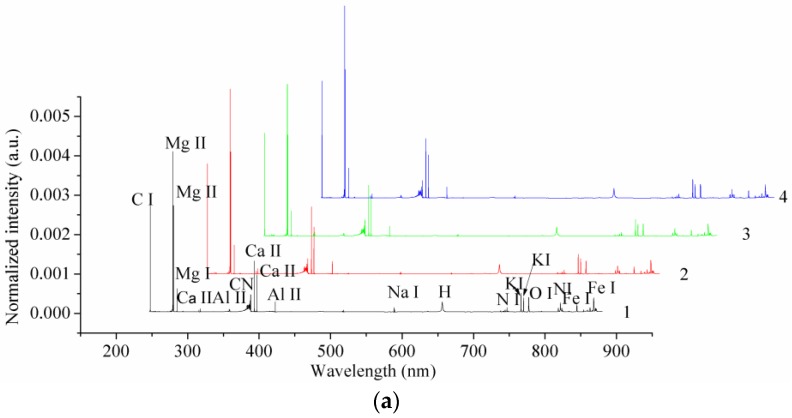

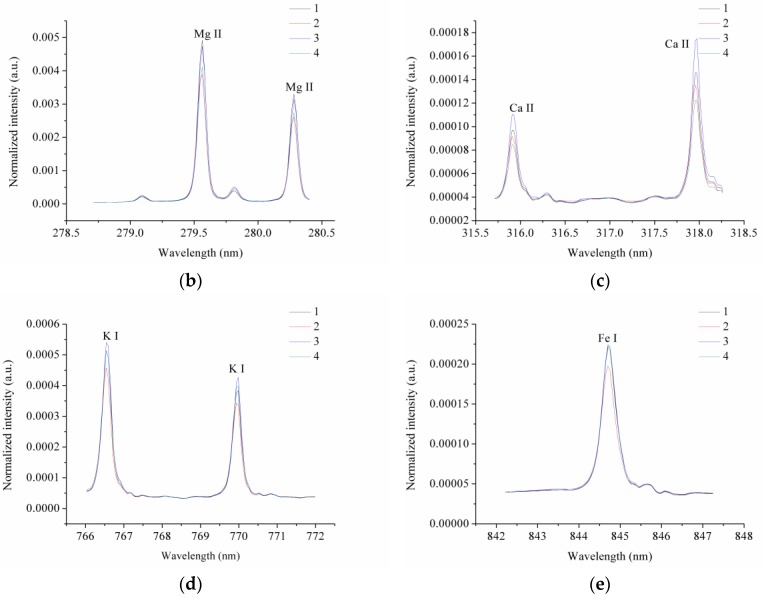
(**a**) The average laser-induced breakdown spectroscopy (LIBS) spectra of four different coffee varieties, and some typical peaks of (**b**) Mg II, (**c**) Ca II, (**d**) K I, and (**e**) Fe I. (a.u.: arbitrary unit).

Figure 2a shows the preprocessed LIBS spectra of one randomly selected sample from each variety. We were able to assign the main elements we observed in the LIBS spectra to C I, Mg I, Mg II, Al II, CN, H, Ca II, Fe I, K I, Na I, N I, and O I. We noted that there were big differences in the spectral intensities of different peaks, of up to 10^6^, and that some of the small peaks were not observable in Figure 2a. We found that the LIBS spectra of the four coffee varieties were similar, with differences in their spectral intensities. Figure 2b–e show some typical peaks of Mg II, Ca II, K I, and Fe I. In observing these peaks, we identified intensity differences for the different coffee varieties.

### 3.3. Principal Component Analysis

**Figure 3:**
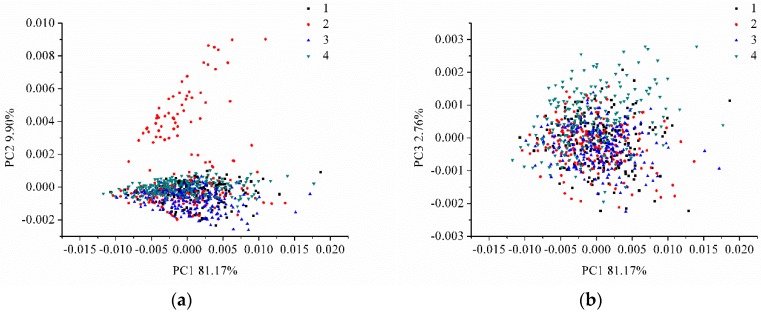

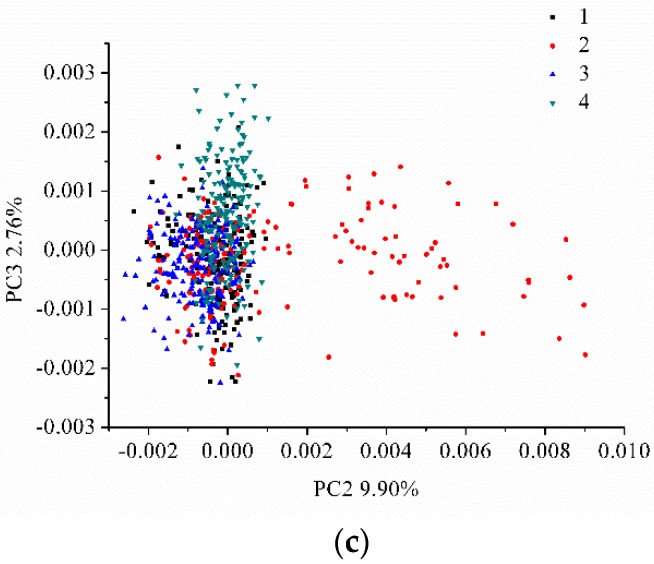
The scores scatter plot of (**a**) PC1 vs. PC2, (**b**) PC1 vs. PC3, and (**c**) PC2 vs. PC3, for the four coffee varieties. (PC: principal component).

A PCA analysis was performed on the LIBS spectra data (20,937 variables). The first 3 PCs explained 81.17%, 9.90%, and 2.76% of the total variance, respectively. The scores scatter plot of PC1 vs. PC2, PC1 vs. PC3, and PC2 vs. PC3 are shown in Figure 3. Here, we noticed that the samples from different varieties could group together, and that they overlapped and were difficult to distinguish from each other. The identification of coffee varieties required further analysis.

### 3.4. Classification Models on the Full LIBS Data

The samples for each variety were randomly divided into calibration and prediction sets at a 2:1 ratio. We used PLS-DA, RBFNN, and SVM to build classification models on the full LIBS data (20,937 variables). PLS-DA, RBFNN, and SVM were able to deal efficiently with a large amount of data. The results of the PLS-DA, RBFNN, and SVM models are shown in Table 1.

The PLS-DA classification result was acceptable, with a classification accuracy of 95.70% and 65.10% for the calibration and prediction sets, respectively. The RBFNN and SVM models obtained better results than the PLS-DA model, yielding in both cases a classification accuracy of 100% for the calibration set and of over 80% for the prediction set. The results of the PLS-DA, RBFNN, and SVM models on the full LIBS spectra indicated that it was feasible to use LIBS spectra with chemometric methods as a way of identifying coffee varieties. Each cultivar’s classification performance was different depending on the discriminant model used.

### 3.5. Analysis of Principal Component Analysis Loadings

The full LIBS spectra contained 20,937 variables; some of these (peaks) corresponded to the elements, while most were background information (useless information). Thus, it was important to select the useful variables for coffee-variety identification and to remove the useless background information. PCA loadings is an effective method for identifying useful information [42]. The first few PCs contained the most useful information, and loadings of each PC indicated the importance of the wavelengths. LIBS is a technique that identifies the elemental composition of the samples, as the qualitative and quantitative analyses of the LIBS spectra focus on the spectral emission of elements. As presented in Figure 3, the first three PCs accounted for more than 93% of total variances; we used loadings of these first three PCs to identify important wavelengths.

**Figure 4:**
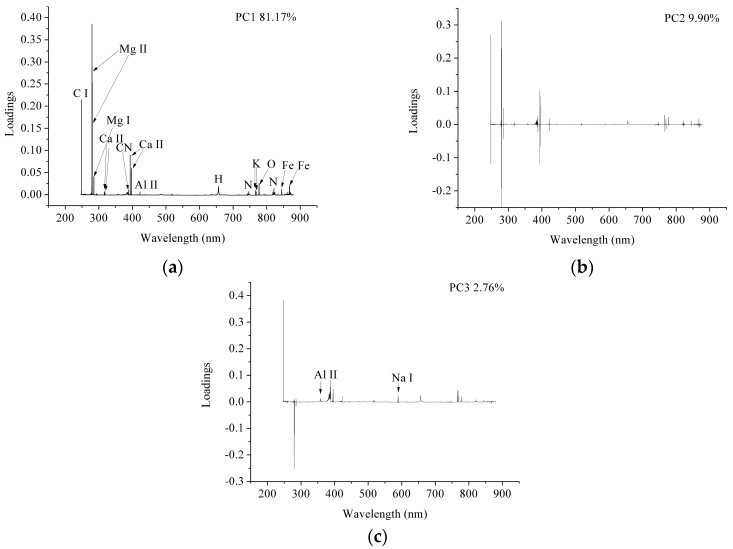
The loading plots of the first three PCs, (**a**) PC1, (**b**) PC2, and (**c**) PC3 for the 4 coffee varieties.

Figure 4 shows the loading plots of the first three PCs using LIBS data from the calibration set. Wavelengths with higher loadings were selected and marked in Figure 4. We noted that, on the peaks, the loading plot correlated well with the LIBS spectra. We were able to assign the selected wavelength variables in Figure 4a to C I, Mg I, Mg II, Al II, CN, H, Ca II, Fe I, K I, N I, and O I, while the selected wavelength variables in Figure 4c were matched to Al II and Na I. Table 2 shows the selected spectral lines and their corresponding elements. The unmarked peaks in Figure 4b,c were on both sides of the corresponding peaks in Figure 4a due to the fact that there were data points (which were not peaks) on both sides of the major peaks with high-emission intensity.

### 3.6. Classification Models on Selected Wavelengths

We used PLS-DA, RBFNN, and SVM to build calibration models for the 20 selected wavelength variables. The results are shown in Table 3. The classification accuracies of the PLS-DA model were 56.77% and 57.81% for the calibration and prediction sets, with 9 LVs. These results were unsatisfactory. The RBFNN and SVM models performed better, both having a classification accuracy of over 80% for their prediction sets. The results indicated the feasibility of identifying coffee varieties by using spectral peaks that correspond to the elements. Each variety’s classification differed depending on the results of the given discriminant model.

## 4. Discussion

The main goal of this study was to identify coffee varieties by using LIBS with chemometric methods. To achieve this goal, four different coffee varieties were collected and measured with a LIBS system developed in our laboratory. In this study, we conducted a simple procedure that included spectral preprocessing, outlier discarding, feature selection, and model establishment.

We used WT to reduce spectral noise. As the analysis of LIBS spectra involved the identification of spectral peaks representing the elements, it was quite important to suppress LIBS spectra noise while keeping the useful information. However, the LIBS spectra could simply be divided into two parts: the background information and the spectral peaks corresponding to the elements. For some of the peaks, the spectral intensity was much higher than the background information, while for others the spectral intensity was only a little higher. One should take care when reducing noise to avoid the elimination of unobvious spectral peaks; WT has proved to be efficient in studies that focused on LIBS [35,36], and it performed well in this study. However, we observed in Figure 1 that the intensity of the peaks dropped as well. The results showed that although WT can be used to reduce noise, studies should also focus on improvements that would aim to reduce noise while keeping peak intensities. Generally, moving average (MA) smoothing and Savitzky-Golay (SG) smoothing have been used as denoising methods in near-infrared spectroscopy and mid-infrared spectroscopy [43,44]. However, to our knowledge, very few studies have looked into their use as a means of preprocessing LIBS spectra. In a future study, we will examine the use of MA and SG to reduce LIBS-spectra noise. The acquisition of LIBS spectra was affected by many factors, and the spectra of different sampling points showed great variations. Preprocessing LIBS spectra could help reduce the influences caused by instruments, samples, and the atmosphere during spectra acquisition. Equally, methods to reduce influences during spectra acquisition should also be studied as a way of improving the identification of coffee varieties.

The full LIBS data contained 20,937 variables, which is quite substantial. According to previous studies [45,46], the coffee varieties could be determined via the concentrations of different elements. The full spectrum contained all of the sample’s elemental information, which was used for coffee-variety identification. As mentioned above, the LIBS data contained the background information and the spectral peaks that corresponded to the elements. The background information may have interfered with the performance of the chemometric methods, increasing the computational task and model complexity. The PCA loading plots in Figure 4 also indicate that the spectral peaks had a larger influence than the background information. The performances of the classification models using full spectra pointed to the effectiveness of spectral line selection. The models that used full spectra performed slightly better than those that used the selected spectral lines. The results indicated that the background information influenced the model performances. The reason for this might be that the background information, such as matrix effects, was generated via samples during the spectra acquisition procedure; these samples might be different due to the different variety samples, and the classification models might therefore perform better with all the variables rather than with the spectral peaks. A similar phenomenon, where classification models using full spectra performed better, can be found in the literature [47]. Moreover, in other studies, when in order to build classification models researchers manually selected spectral regions with spectral peaks instead of the full spectra and spectral peaks, they obtained satisfactory results. It should be noted that in these regions there was also background information [48,49,50]. On the other hand, Vors et al. have shown that optimal data preprocessing and variable-selection methods have a great influence on classification performances [51]. Some other studies have also indicated that these are also influenced by the selection of optimal variables [52,53,54]. It was therefore important to select variables or regions that were related to the elements that contribute the most to coffee-variety identification. This procedure helps significantly reduce the number of variables and the computation task, and simplifies the model. In our study, for example, the number of variables dropped from 20,937 to 20, resulting in a 99.90% reduction; the classification performances differed only slightly. The computation of the SVM and RBFNN models using the full-spectra calibration set (768 × 20,937) takes more than 12 h on a personal computer (computer hardware: CPU: Intel Core (TM) i7-6700; RAM: 16 GB; Graphics card: NVIDIA GeForce GTX 750 Ti; 256 GB solid state disk). By using selected wavelengths, this computation task was significantly reduced from hours to minutes. Future studies should also explore the methods used to evaluate the importance of spectral peaks (elements) in LIBS data analysis.

The models using the selected wavelength variables performed worse than the models on the full LIBS data. The SVM and RBFNN models performed better than the corresponding PLS-DA models. The nonlinear neural networks (RBFNN and SVM) showed better results than the linear PLS-DA models. The reason might be attributable to the nonlinear effects in the LIBS data [55]. The overall results indicate that LIBS, combined with chemometric methods, could be used for coffee-variety identification. SVM and RBFNN showed some advantages over the linear PLS-DA models. However, studying more samples and more chemometric methods should help obtain better identification results.

## 5. Conclusions

We used LIBS in combination with chemometric methods, including discriminant models and variable selection, to identify coffee varieties. Preprocessing the LIBS spectra using WT effectively reduced spectral noise. Via PCA loadings we selected variables corresponding to C I, Mg I, Mg II, Al II, CN, H, Ca II, Fe I, K I, Na I, N I, and O I as important variables for identifying coffee varieties. PLS-DA, RBFNN, and SVM models, built by using full spectra and important variables, showed acceptable results, indicating the effectiveness of chemometrics in LIBS analysis. The overall results pointed to the feasibility of identifying coffee varieties by using LIBS with chemometric methods. For future studies, more samples are needed in order to produce more robust classification models. Additionally, chemical analyses of elements in coffee beans should be conducted for further proof of this method’s effectiveness. Further research should also focus on the usage of different methods as a way of selecting the elements that contribute most to classification. This study provides an example of a qualitative analysis of LIBS data with chemometric methods for researchers conducting further studies. Our attempt to select the most contributed variables (elements) in LIBS spectra should also help with the development of on-line qualitative analysis systems.

## Figures and Tables

**Table 1:** The results of the partial least squares-discriminant analysis (PLS-DA), radial basis function neural network (RBFNN) and support vector machine (SVM) models on the full LIBS data.

Models	^a^ LVs/Spread/(C, γ)	Calibration Accuracy (%)					Prediction Accuracy (%)				
		1	2	3	4	Total	1	2	3	4	Total
PLS-DA	20/*/*	95.83	98.96	96.35	91.67	95.70	42.71	82.29	75.00	60.42	65.10
RBFNN	*/100/*	100	100	100	100	100	82.29	66.67	80.21	98.96	82.03
SVM	*/*/(9.1896, 0.0039)	100	100	100	100	100	68.75	80.21	90.63	94.79	83.59

^a^ LVs are the number of latent variables in the PLS-DA model; spread is the spread value in RBFNN; nodes are the number of nodes in the hidden layer of SVM; * means that no parameters exist for the given model.

**Table 2:** The spectral lines selected by PCA loadings and the corresponding element assignments.

Wavelength (nm)	Element	Wavelength (nm)	Element	Wavelength (nm)	Element	Wavelength (nm)	Element
247.87	C I	317.97	Ca II	422.68	Al II	769.98	K I
279.56	Mg II	358.57	Al II	588.99	Na I	777.33	O I
280.28	Mg II	388.32	CN	656.38	H	821.69	N I
285.22	Mg I	393.39	Ca II	746.92	N I	844.75	Fe I
315.93	Ca II	396.85	Ca II	766.54	K I	868.10	Fe I

**Table 3:** The results of the PLS-DA, RBFNN and SVM models on selected wavelength variables.

Models	LVs/Spread/(C, γ)	Calibration Accuracy (%)					Prediction Accuracy (%)				
		1	2	3	4	Total	1	2	3	4	Total
PLS-DA	9/*/*	29.30	89.06	56.77	51.56	56.77	14.58	97.92	66.67	52.08	57.81
RBFNN	*/0.2/*	80.73	77.60	89.06	100	86.85	56.25	77.08	87.5	100	80.21
SVM	*/*/(84.4485, 0.0359)	74.48	67.71	90.10	98.44	82.68	46.88	84.38	90.63	100	80.47
